# New York Medical Independent

**Published:** 1864-04

**Authors:** 


New York Medical Independent.—We have the pleasure of adding to our exchange list the *New York Medical Independent*, a brief prospectus of which will be seen by reference to our advertisement sheet. We bid the new journal "God-speed" in every honest endeavor to advance the true progress of medical science. The first paragraph of the Editorial Introductory is a little ambiguous; and we suggest that the desire to reform, advance and improve may become revolutionary unless properly controlled. Independent means a great many things, and if we were not well acquainted with the projectors of the enterprise, we should be looking for something radical in medical practice. As it is, we expect nothing more "than is in the bond,"—a first-rate weekly medical journal, worthy the support of those who have at heart the true interests of medical science. The first number shows vigor and strength, and we hope it may accomplish all its objects. We have re-produced one of its first articles, and hope notice will be taken of the selection—"*McMunn's Elixir of Opium.* Has it any Superiority over other Preparations?"

